# Extra-Uterine Pregnancy—Removal of the Child by the Cæsarean Operation

**Published:** 1845-11

**Authors:** M. M’Culloch

**Affiliations:** Lecturer on Midwifery, M’Gill College, Montreal


*Extra Uterine Pregnancy.—Removal of the Child by the Cæsarean Operation.* By M. M'Culloch, M. D., Lecturer on Midwifery, M'Gill College, Montreal.—Madame Reaume, aged 21, a native of St. Eustache, in this District, had, on a former occasion, a living child at the full term, and when she first consulted me, in May, 1828, was again pregnant, and had passed about fifteen months from the last catamenial period, and about ten months from the time of quickening, without having experienced any symptom of parturition, although the abdominal enlargement and other symptoms left no doubt of the existence of pregnancy. During the first nine months of that period, she frequently experienced severe pains in the right iliac region; but after the completion of the usual term of utero-gestation the motion of the child was no longer felt, and she thought her sufferings, in consequence, became much less severe. About the same time the milk began to flow from her breasts, and a lochial discharge appeared, and continued several days. Notwithstanding this remarkable change the abdomen did not decrease, and the fluctuation only of an immense quantity of fluid could be detected.

She remained nearly in the same condition until the following month of November, when I endeavoured, by tapping the abdomen, to alleviate her sufferings, and about thirty-six pints of liquid, of the colour and consistence of pale ink, were drawn off. The child could now, for the first time, be distinctly felt, under the integuments, and the position of its body and limbs easily traced. She experienced no inconvenience or bad consequences from the operation, although she had very imprudently ventured to walk a few hundred yards to the village Church on the following day. Diuretics, and occasionally a purgative, were for some time administered, and I had the satisfaction to find that she afterwards remained free from the slightest symptom of dropsy. She, nevertheless, continued to suffer daily from fever and debility until the month of June following, when putrid matter, mixed with quantities of hair from the child's head, began to ooze from her navel. The skin was inflamed a few inches round an opening that would admit the point of the finger, and nature was, in this way, evidently making a most interesting effort to expel the child, and save the life of the mother; but she had become so feeble and emaciated as scarcely to leave a chance of her surviving a few days, and I thought I was, under the circumstances, warranted in proposing the Cæsarean operation as her only hope. At the same time, her alarm was much increased by observing two worms escaping from the navel, and she, without hesitation, agreed to submit to whatever I thought would afford her a chance of recovery. Being then six miles distant from the nearest professional friend, I did not, under the circumstances, consider myself warranted in waiting for assistance; I therefore had her at once placed on a table, and made an incision in the linea alba extending five or six inches downwards from the navel, and in the third year of her pregnancy, removed a putrid child of the ordinary size at birth. She did not lose an ounce of blood, and bore the operation with great courage. No vestige of a placenta remained, and the child was found, in a sac that had formed adhesions all round to the walls of the abdomen, and appeared to be the fallopian tube enormously distended and thickened. It contained, besides the child, a quantity of very offensive matter. Nearly all the bones of the toes and fingers were found detached, and some of them adhering to the sides of the cavity were carefully removed; a small tent was then placed at the bottom of the incision to favor the escape of matter, and its edges were kept in contact with adhesive plaster, supported with a bandage. She afterwards continued to improve daily, although the thermometer, at the time of the operation and for several days after, was upwards of 90° in the shade. Her progress, notwithstanding, from a state of extreme prostration to perfect health was so rapid, that she was able, without inconvenience, to be taken six miles to church a month after the operation.

She has since enjoyed excellent health, and, without regret, remains childless.—*Brit. Am. Journ. of Med. and Phys. Science.*

*M. Flourens on the Development of Bone.*—The following three propositions embody the chief results of M. Flourens' recent investigations upon this most interesting subject of enquiry.

1. *Bone is formed by the periosteum.* 2. *It grows by the superposition of external layers.* 3. *The medullary canal is enlarged by the absorption of the internal layers of the bone.*

The experiments, on which the first of these propositions is based, were performed on dogs. A portion of one of the ribs was excised; removing only the bone, and leaving the periosteum behind. It was found that, after the expiry of a few days, a minute osseous nucleus was formed within the periosteum between the two divided ends of the rib. This nucleus became larger and larger, until at length it rejoined these ends, the one to the other, thus filling up the void space between them.

The numerous preparations, exhibited by M. Flourens at the Royal Academy, clearly show that the new bone is formed in the periosteum; that, when it is first formed, it is completely insulated and apart from the old bone; and that it is only by its gradual development and extension that it ultimately reaches the two divided ends of the old bone, thus re-uniting them together.

The *second* proposition—*bone grows in size by the super-position of external layers*—was established by numerous experiments on dogs and rabbits. One of the tibiæ was exposed, the periosteum divided, and a ring of platinum wire was then passed around between the bone and its investing membrane. The wound being then closed and left undisturbed, it was found after a certain period that the new bone, that had been formed, fairly invested with its recently-deposited layers the platinum wire. The *third* proposition was equally satisfactorily made out by the preparations that were exhibited.—*Med. Chirurg. Review from Compt. Rend.*

